# Paralysis caused by dinotefuran at environmental concentration via interfering the Ca^2+^–ROS–mitochondria pathway in *Chironomus kiiensis*

**DOI:** 10.3389/fpubh.2024.1468384

**Published:** 2024-10-02

**Authors:** Fenghua Wei, Weiwen Gu, Fengru Zhang, Shuangxin Wu

**Affiliations:** ^1^School of Chemistry and Environment, Jiaying University, Meizhou, China; ^2^School of Physics and Electrical Engineering, Jiaying University, Meizhou, China

**Keywords:** neonicotinoid insecticides, long-term exposure, mitochondria, Chironomidae, environmental dose

## Abstract

**Introduction:**

Dinotefuran as the third-generation of neonicotinoid insecticides is extensively used in agriculture worldwide, posing a potential toxic threat to non-target animals and humans. However, the chronic toxicity mechanism related to mitochondria damage of dinotefuran to non-target animals at environmental concentration is unclear.

**Methods:**

In this study, the mitochondria damage and oxidative stress of dinotefuran on *Chironomus kiiensis* were investigated at environmental concentrations by long-term exposure. At the same time, relevant gene expressions of these toxicity indexes were measured as sensitive ecotoxicity biomarkers to reflect the toxic effects of dinotefuran on Chironomidae.

**Results:**

Our present study showed that chronic exposure to environmental concentrations of dinotefuran resulted in behavioral inhibition in the larvae of Chironomidae. For burrowing inhibition of 10 days, the lowest observed-effect concentration (LOEC) and 50% inhibitory concentration (IC_50_) were 0.01 (0.01–0.04) and 0.60 (0.44–0.82) μg/L, respectively. Dinotefuran promoted the release of intracellular calcium ions (Ca^2+^) in Chironomidae via dysregulating the gene expressions of *atp2b*, *camk ii*, and *calm*. Subsequently, the disruption of the Ca^2+^ signaling pathway induced oxidative stress by raising reactive oxygen species (ROS), hydrogen peroxide (H_2_O_2_), and malonaldehyde (MDA) levels. Thus, the over-release of Ca^2+^ and ROS disordered the normal functioning of mitochondrial-related pathways by dysregulating the expressions of mitochondria-related genes of *atpef0a*, *sdha*, and *cyt b*.

**Conclusion:**

Our findings showed that low environmental concentrations of dinotefuran caused paralysis of the midge via interfering the Ca^2+^–ROS–mitochondria pathway. These results provided data support for assessing the potential environmental risk of dinotefuran.

## Graphical abstract

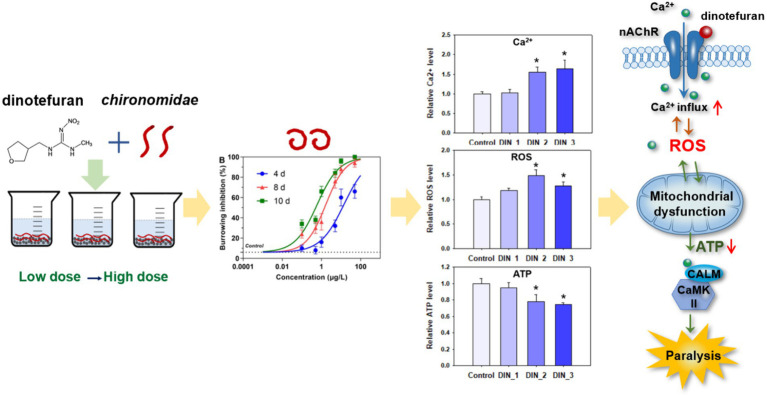

## 1 Introduction

Neonicotinoid insecticides are the fastest-growing systemic pesticides in the world due to being considered to be less toxic to mammalian species than traditional insecticide classes, such as organochlorines and organophosphates (1). Dinotefuran, which is a third-generation neonicotinoid insecticide, has a tetrahydrofuran ring but no halogen elements of other neonicotinoids (2). It has more excellent properties than first-and second-generation neonicotinoids, such as higher insecticidal activity, quicker uptake, smaller resistance, broader spectrum, and safer for the environment and humans (3). Nowadays, dinotefuran has been widely used, accounting for more than 25% of the global pesticides used (4). Excessive use and high water solubility of dinotefuran unavoidably remained in residues in surface waters, causing harm to aquatic organisms and humans (5).

Dinotefuran was less studied in previous reports compared with the other neonicotinoid insecticides. A few studies reported the detection of waterborne dinotefuran in various regions. Xiong et al. (6) detected neonicotinoid insecticides from a paddy field to receiving waters in the Poyang Lake basin of China, showing that dinotefuran was the dominant neonicotinoid with a mean concentration of 200 ± 296 ng/L and the maximum concentration of 802 ± 139 ng/L. Dinotefuran was detected with a concentration of 12.7–75.5 ng/L in rivers near maize fields in Ontario, Canada (7) and 1.60–134 ng/L in streams across the United States (8). Putri et al. (9) analyzed neonicotinoid occurrence in tropical environmental waters of Indonesia, the highest concentration of dinotefuran was 23.12 ng/L in estuaries and mangrove areas. Thompson et al. (10) investigated neonicotinoid insecticides in well tap water and human urine samples in eastern Iowa, the max concentration of dinotefuran was 3.9 ng/L in groundwater samples and 2.9 μg/g in urine samples.

It is well known that the toxicity target of neonicotinoids is the nicotinic acetylcholine receptors (nAChRs) of insects. They act on nAChRs and disrupt the central nervous system of insects, thus, insects become paralyzed and even die due to overexcitement (11). Even so, numerous recent reports have found neonicotinoids could cause unintended toxic effects on non-target organisms, even humans. Therefore, the exploration of their additional toxic mechanism has become the emerging focus of public attention. Although the toxicity of dinotefuran is low, the toxicity to non-target organisms cannot be ignored. Liu et al. (12) showed that more than 1.0 mg/kg of dinotefuran caused oxidative stress and genetic toxicity in earthworms (*Eisenia fetida*) during the 28 d exposure. Dinotefuran (0.1, 0.5, and 2.0 mg/L) induced oxidative stress and DNA damage in juvenile Chinese rare minnows (*Gobiocypris rarus*) after 60 d exposure (13). Though these toxicities of dinotefuran have been studied in some organisms, little is known about Chironomidae, especially for environmental concentrations and long-term exposure. Chironomid larvae are the main invertebrates in freshwater ecosystems and play important ecological functions because they are natural baits for many other aquatic organisms (14). In addition, Chironomidae are more sensitive to neonicotinoids compared with a lot of other aquatic invertebrates (5). Therefore, it is necessary to study the chronic toxicity mechanism of dinotefuran to Chironomidae as representative organisms at environmental concentrations. In addition, as the experimental animal chironomid is a lower invertebrate and belongs to the class of insects of the invertebrate phylum, therefore no ethical review is required.

In this study, *Chironomus kiiensis* was chosen as the test organism. Compared to the commonly used *Chironomus dilutus* (approximately 60 days), the life cycle of *Chironomus kiiensis* is shorter, only approximately 23 days, which is more time-saving on biological tests. The mitochondria damage and oxidative stress of dinotefuran on *Chironomus kiiensis* were investigated at environmental concentrations by long-term exposure. At the same time, relevant gene expressions of these toxicity indexes were measured as sensitive ecotoxicity biomarkers to reflect the toxic effects of dinotefuran on Chironomidae. These results will improve our understanding of the potential toxic mechanisms of dinotefuran to Chironomidae and provide data support for assessing neonicotinoid insecticides’ potential environmental risks.

## 2 Materials and methods

### 2.1 Materials

Dinotefuran (CAS: 165252-70-0), thiamethoxam-*d*_3_ (internal standard), and imidacloprid-*d*_4_ (surrogate standard) were purchased from Dr. Ehrenstorfer GmbH (Augsburg, Germany) with purity >98%. The midges, *Chironomus kiiensis* (*C. kiiensis*) were cultured in Jiaying University according to the standard protocol of USEPA2000 proposed by the U.S. Environmental Protection Agency.

### 2.2 Toxicological assay

Test water was freshly prepared by a range of concentrations (0.1, 0.5, 1, 5, 10, and 50 μg/L) of dinotefuran (DIN_1, DIN_2, DIN_3, DIN_4, DIN_5, and DIN_6) into reconstituted moderately hard water. Negative control and solvent control were tested in the meantime. A 0.5 cm layer of quartz sand and 200 mL of testing solution were introduced into each 500 mL beaker. Ten 1st instar larvae of *C. kiiensis* were randomly added into each beaker with 3–5 replicates per treatment group or control group. The entire exposure period ended until the first pupa appeared (approximately 10 days). The test solution was changed once on day 5. The organisms were fed ground fish food every 2 days per beaker. Water quality parameters (i.e., conductivity, pH, temperature, and dissolved oxygen) in the test solution were monitored every day and ammonia nitrogen was monitored on days 0, 5, and 10. At the end of the exposure period, the survival larvae were evaluated for a series of toxicity indexes, including lethality, burrowing inhibition, cellular responses, and the corresponding gene expressions. For burrowing inhibition, under normal circumstances, a larva of Chironomidae burrows into sand for nesting. If more than half of its body fails to burrow successfully, burrowing behavior is considered inhibited. The exposed solution was sampled at 5 and 10 days in three replicates and analyzed for dinotefuran actual concentrations using HPLC-MS/MS following a previously developed method by Wei et al. (15). More details on the quantification of dinotefuran are shown in the Supplementary material and qualification parameters for the analyte are listed in Supplementary Table S1.

### 2.3 Intracellular calcium ion level

Survival organisms in each group of control and three treat groups (DIN_1, DIN_2, and DIN_3) were used to measure the content of the intracellular Ca^2+^. The concentration of Ca^2+^ was measured using a Fura-2/AM probe (Beyotime, Haimen, China). Survival midge larvae were homogenized in 3 mL of phosphate buffer solution (PBS) using a glass homogenizer and sieved with a 75 μm cell strainer. The homogenate was centrifuged twice for washing purposes at 1000 g and 4°C. The cell precipitate was resuspended in PBS. The cell suspension was preloaded with 2 μmol/L Fura-2/AM at 37°C for 30 min and was centrifugated at 1000 g for 5 min after being washed in PBS solution. Then the cells were resuspended in PBS and moved into 6-well plates (1 × 10^5^ cells/well). The fluorescent signal was measured using a microplate spectrophotometer (Biotek, Synergy H1, United States) with emission wavelength at 510 nm and excitation wavelength at 340 and 380 nm. The relative amount of Ca^2+^ was calculated as the ratio of F340/F380 relative to control.

### 2.4 Oxidative stress indexes

Survival larvae in control and three treat groups (DIN_1, DIN_2, and DIN_3) were homogenized in 1 mL PBS for 3 min and the solution was centrifuged at 10,000 g at 4°C for 10 min. The supernatants were used for measuring protein content, hydrogen peroxide (H_2_O_2_) concentration, and malondialdehyde (MDA) content using commercial assay kits according to the manufacturer’s protocols (Beyotime).

For H_2_O_2_ levels, 50 μL of supernatant sample or standard was added to the test well followed by 100 μL of H_2_O_2_ detection reagent to each well. The mixture solution was gently shaken and remained at room temperature for 30 min and immediately determined 560 nm. The concentration of H_2_O_2_ was calculated according to the standard curve.

For the MDA content, 100 μL of the standard products with different concentrations or 100 μL of the supernatant sample were added to the centrifuge tube. Then 200 μL of MDA detection fluid was added. The mixture was heated in a boiling water bath for 15 min and cooled to room temperature. After centrifugation at 1000 g for 10 min at room temperature, 200 μL of supernatant was added to the 96-well plate, and then the absorbance was measured at 532 nm.

ROS levels were measured using a commercial ROS assay kit (Beyotime) following the manufacturer’s protocol. Surviving midge larvae were cut into small pieces with scissors. The fragment of tissue was gently rubbed on the 300 mesh nylon net which was put on the small test tube. Cell suspension was collected, and centrifuged at 500 g for 10 min, then the supernatant was removed. The cell precipitation was washed with PBS 1–2 times. The cell suspension was suspended in the DCFH-DA probe and incubated in a cell incubator at 37°C for 20 min. Subsequently, the mixture was inverted and mixed every 3–5 min so that the probe was in full contact with the cells. The cells were washed three times with serum-free cell culture solution to fully remove DCFH-DA that did not enter the cells. The concentration of ROS was detected at 488 nm excitation wavelength and 525 nm emission wavelength.

### 2.5 Mitochondria indexes

Mitochondrial membrane potential (MMP) was measured using a commercial MMP assay kit with JC-1 (Beyotime) following the standard procedure by the manufacturer’s protocols. Mitochondrial depolarization was measured by the relative ratio of red to green fluorescence on a multifunctional microplate reader. Red fluorescence was detected at excitation light 525 nm and emission light 590 nm. Green fluorescence was detected at excitation light 490 nm and emission light 530 nm.

Adenosine triphosphate (ATP) level was measured using a commercial ATP assay kit (Beyotime) according to the manufacturer’s protocols. A total of 100 μL of ATP test fluid was added to the test tube, which was placed at room temperature for 3–5 min, so that all the background ATP was consumed, thereby reducing the background. A total of 20 μL of sample or standard was introduced in the detection tube and mixed quickly, and the response by the chemiluminescence mode was measured after at least 2 s.

### 2.6 Measurements of gene levels

Survived larvae (DIN_1, DIN_2, and DIN_3) were immediately frozen with liquid nitrogen before use. Total RNA was isolated using an RNeasy Mini Kit (Qiagen, Hilden, Germany) according to the manufacturer’s protocol. Expressions of 9 genes (Supplementary Table S2) were quantified using a real-time quantitative polymerase chain reaction (RT-qPCR) according to the method of the previous study by Wei et al. (15). In brief, β-actin was chosen as an internal control. The RNA samples were reversely transcribed into cDNA by using a Bestar^™^ qPCR-RT Kit (DBI-2220, German). RT-qPCR was performed in an ABI 7500 fluorescence quantitative PCR instrument (ThermoFisher, United States). The fold changes of the target genes were calculated using a 2^−ΔΔCT^ method.

### 2.7 Statistical analysis

The concentration-effect curve was fitted by the GraphPad Prism 5.0 software (San Diego, CA, United States). Differences among the treatments were analyzed with one-way ANOVA by SPSS 17.0 software (SPSS Inc., Chicago, Ill., United States). The *p*-value <0.05 was considered statistically significant.

## 3 Results

### 3.1 Phenotypic toxicity

**Figure 1:**
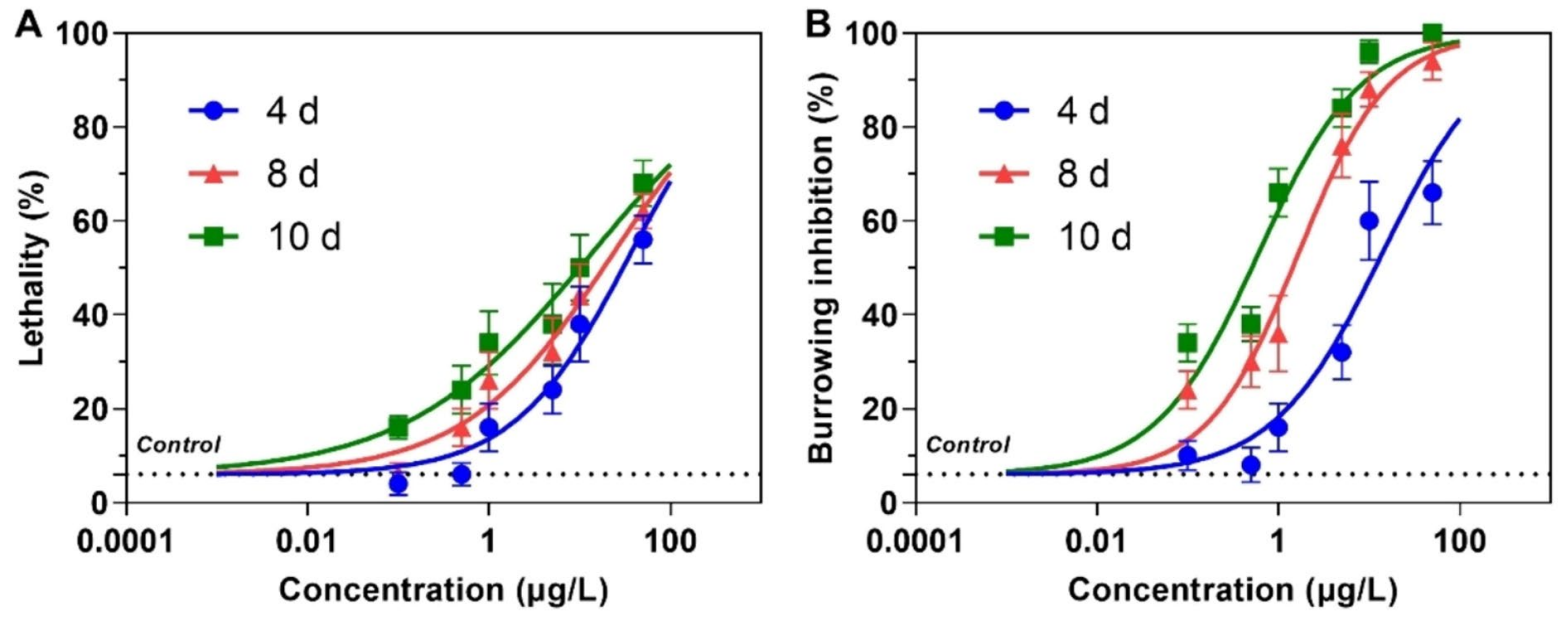
Lethality **(A)** and burrowing inhibition **(B)** of the larva of *Chironomus kiiensis* after exposure to dinotefuran. Data are expressed as mean ± standard error (*n* = 5). The dotted line represents solvent control.

Dinotefuran concentrations in exposure solutions varied little under the experiment duration (Supplementary Table S3). Survival and burrowing behavior of the larva were impaired by dinotefuran in a concentration-dependent manner at 4, 8, and 10 days (Figures 1A,B and Supplementary Table S4). There was no significant difference in larva lethality between negative control and solvent control (Supplementary Figure S1). For lethality of 4 days, the lowest observed-effect concentration (LOEC), and 10 and 50% lethal concentrations (LC_10_ and LC_50_) were 0.46 (0.15–1.61), 1.40 (0.55–3.25), and 36.4 (21.5–61.6) μg/L (mean (95% confidence interval)), respectively (Table 1). For burrowing inhibition of 4 days, LOEC, and 10 and 50% inhibitory concentrations (IC_10_ and IC_50_) were 0.24 (0.07–0.93), 0.66 (0.24–1.72), and 13.9 (8.6–22.5) μg/L, respectively (Table 1). For lethality of 8 days, the LOEC, LC_10_, and LC_50_ were 0.09 (0.02–0.43), 0.38 (0.12–1.06), and 23.3 (13.0–41.6) μg/L, respectively (Table 1). For burrowing inhibition of 8 days, LOEC, IC_10_, and IC_50_ were 0.06 (0.02–0.17), 0.13 (0.06–0.29), and 1.66 (1.16–2.37) μg/L, respectively (Table 1). For lethality of 10 days, the LOEC, LC_10_, and LC_50_ were 0.01 (0.002–0.12), 0.08 (0.02–0.33), and 13.4 (6.7–26.8) μg/L, respectively (Table 1). For burrowing inhibition of 10 days, LOEC, IC_10_, and IC_50_ were 0.01 (0.01–0.04), 0.04 (0.02–0.08), and 0.60 (0.44–0.82) μg/L, respectively (Table 1).

**Table 1:** Effect concentrations (μg/L) of dinotefuran exposed to 1st instar larva of *Chironomus kiiensis* (Data are expressed as mean ± standard error (*n* = 5)).

Toxicity index	Effect concentration	4 d	8 d	10 d
Lethality (mean (95% confidence interval))	LOEC^a^	0.46 (0.15–1.61)	0.09 (0.02–0.43)	0.01 (0.002–0.12)
	LC_10_^b^	1.40 (0.55–3.25)	0.38 (0.12–1.06)	0.08 (0.02–0.33)
	LC_50_^c^	36.4 (21.5–61.6)	23.3 (13.0–41.6)	13.4 (6.7–26.8)
Burrowing inhibition (mean (95% confidence interval))	LOEC	0.24 (0.07–0.93)	0.06 (0.02–0.17)	0.01 (0.01–0.04)
	IC_10_^d^	0.66 (0.24–1.72)	0.13 (0.06–0.29)	0.04 (0.02–0.08)
	IC_50_^e^	13.9 (8.6–22.5)	1.66 (1.16–2.37)	0.60 (0.44–0.82)

a LOEC, lowest observed-effect concentration.

b LC_10_, 10% lethal concentration.

c LC_50_, median lethal concentrations.

d IC_10_, 10% inhibitory concentration.

e IC_50_, median inhibitory concentration.

### 3.2 Intracellular Ca^2+^ concentration

**Figure 2:**
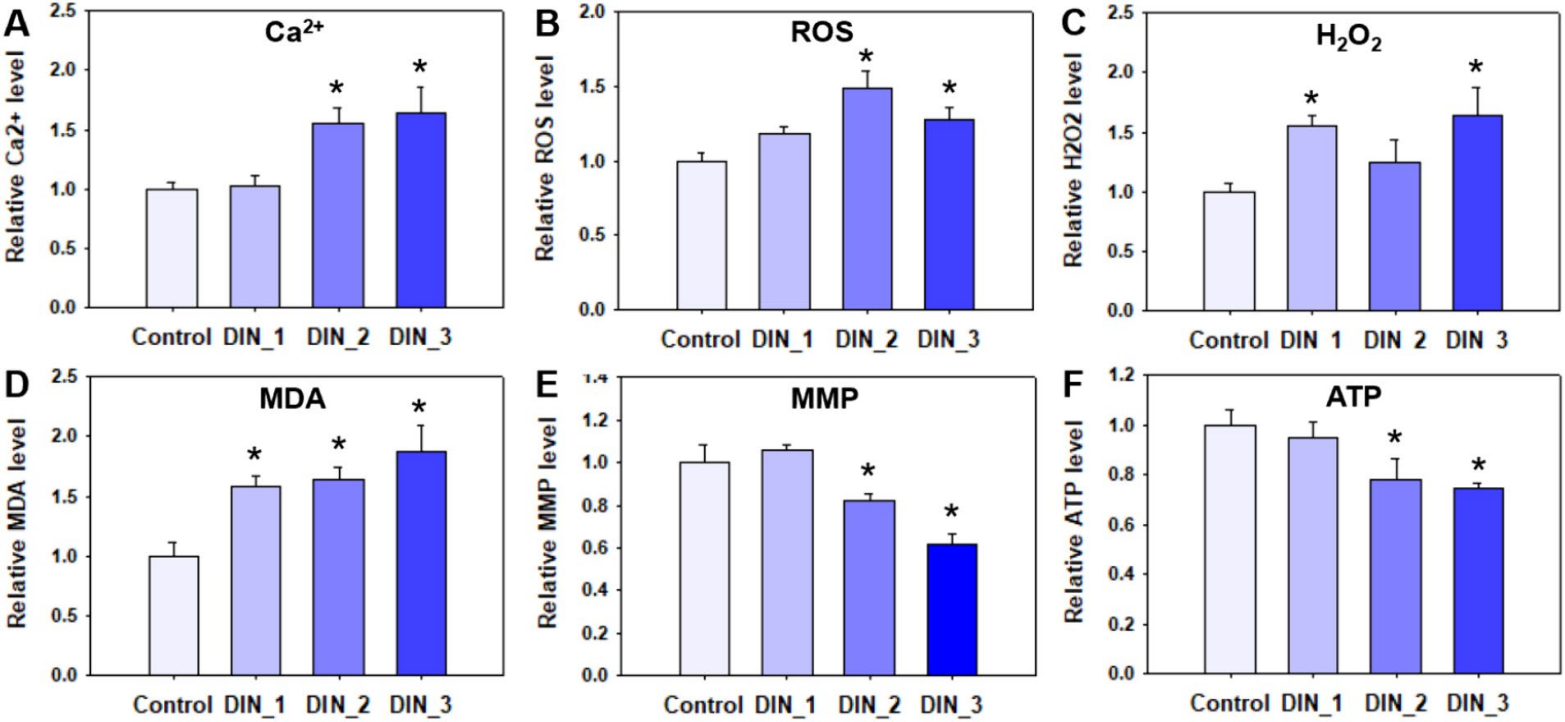
Intracellular calcium ion **(A)**, oxidative stress **(B–D)**, and mitochondria **(E,F)** indexes in 1st instar larva after exposure to dinotefuran until the first pupa appears. Data are expressed as mean ± standard error (*n* = 3). The asterisk denotes a significant difference from the solvent control (*p* < 0.05). ROS, reactive oxygen species; H_2_O_2_, hydrogen peroxide; MDA, malondialdehyde; MMP, mitochondrial membrane potential; ATP, adenosine triphosphate.

**Figure 3:**
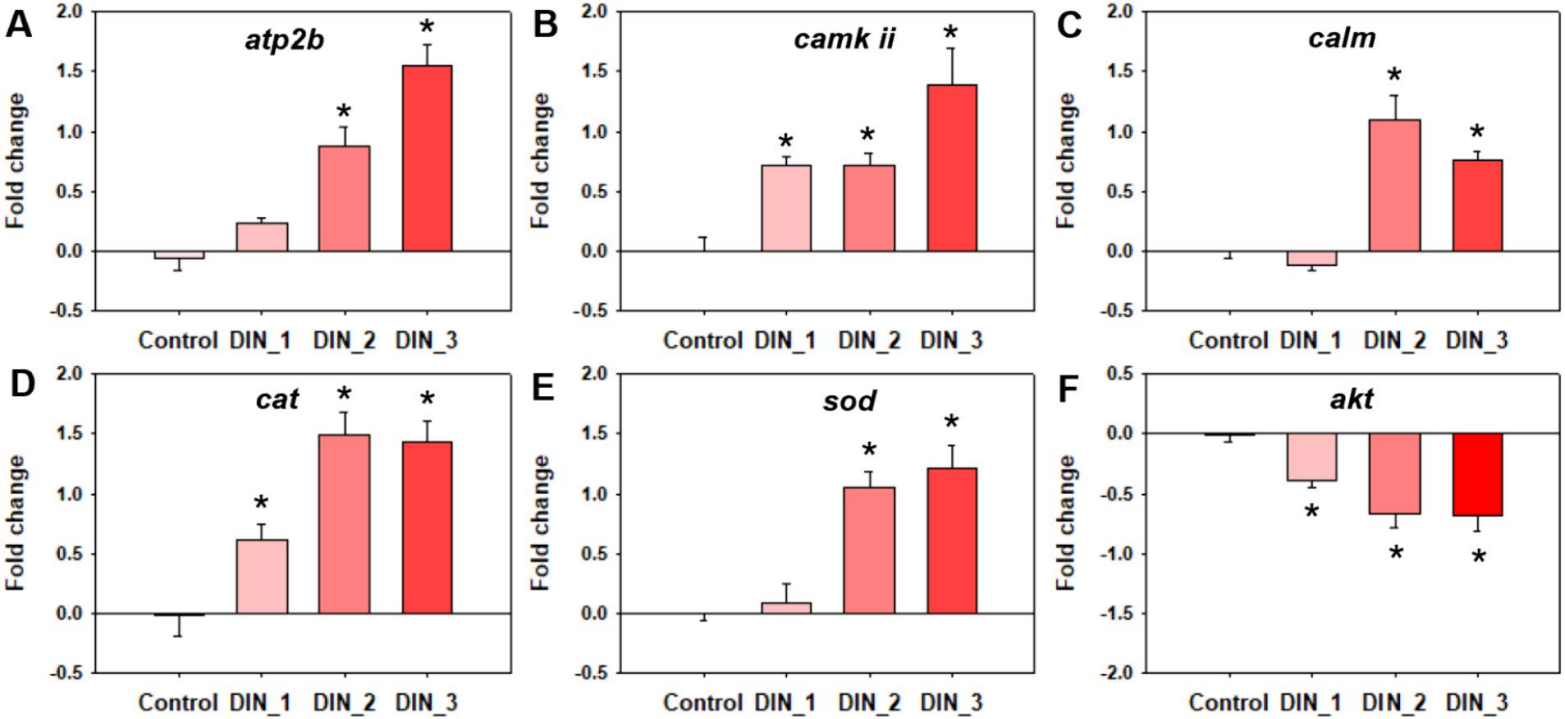
Genes expressions related to calcium ion **(A–C)** and oxidative stress **(D–F)** in 1st instar larva after exposure to dinotefuran until the first pupa appears. Data are expressed as mean ± standard error (*n* = 3). The asterisk denotes a significant difference with *p* < 0.05 compared with the solvent control.

Dinotefuran significantly stimulated the release of intracellular Ca^2+^ concentrations of the midges above concentrations of 0.5 μg/L (DIN_2-DIN_3) after 10 d exposure (*p* < 0.05) (Figure 2A and Supplementary Table S5). The gene expressions of *atp2b* (Ca^2+^ transporting ATPase plasma membrane), *camk ii* (calcium/calmodulin-dependent protein kinase II), and *calm* (calmodulin) related to the calcium pathway were significantly upregulated after exposure to dinotefuran above the concentrations of 0.1 μg/L (DIN_1-DIN_3) or 0.5 μg/L (DIN_2-DIN_3) (Figures 3A–C and Supplementary Table S6).

### 3.3 Oxidative stress

The levels of ROS were significantly increased after 10 d exposure to dinotefuran at 0.5–1 μg/L (DIN_2-DIN_3) in larva (Figure 2B). The H_2_O_2_ levels were significantly upraised at 0.1 and 1 μg/L (DIN_1 and DIN_3) by dinotefuran (Figure 2C) but except for 0.5 μg/L (DIN_2). Similarly, the MDA contents were significantly increased at 0.1–1 μg/L (DIN_1-DIN_3) of dinotefuran (Figure 2D). The gene expressions of *cat* (catalase) and *sod* (superoxide dismutase) related to oxidative stress were significantly upregulated relative to the control group at DIN_1-DIN_3 and DIN_2-DIN_3 groups, respectively (Figures 3D,E). Conversely, the gene expressions of *akt* (RAC serine/threonine-protein kinase) were significantly downregulated at DIN_1–DIN_3 groups relative to the control group (Figure 3F).

### 3.4 Mitochondrial dysfunction

**Figure 4:**
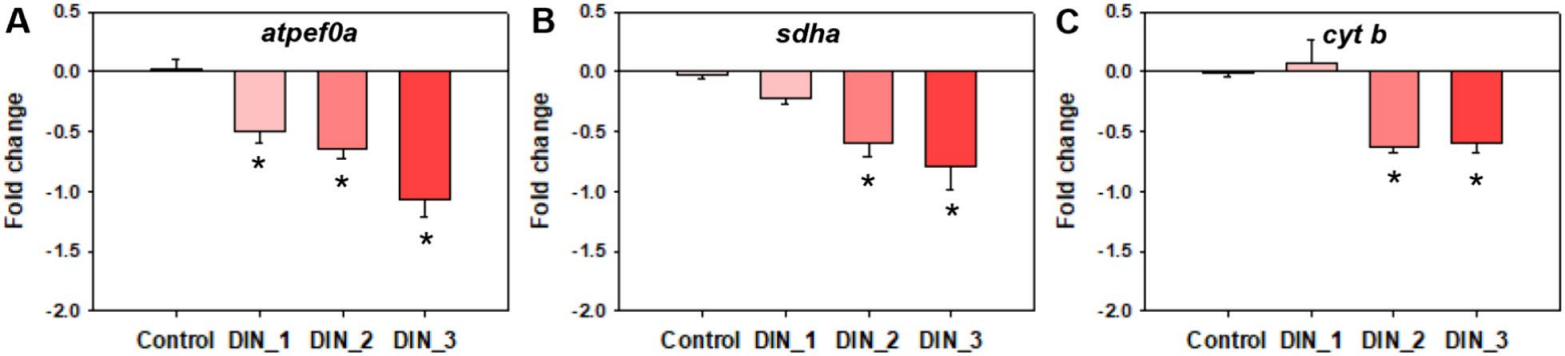
Genes expressions related to mitochondrial function **(A–C)** in 1st instar larva after exposure to dinotefuran until the first pupa appears. Data are expressed as mean ± standard error (*n* = 3). The asterisk denotes a significant difference with *p* < 0.05 compared with the solvent control.

The levels of MMP and ATP were decreased after exposure to dinotefuran (Figures 2E,F). Significant reduction (*p* < 0.05) was observed at concentrations of 0.5 and 1 μg/L (DIN_2–DIN_3). In addition, the related gene expressions of *atpef0a* (F-type H^+^-transporting ATPase subunit a) were significantly downregulated at 0.1–1 μg/L of dinotefuran (DIN_1–DIN_3) (Figure 4A). Other important genes, *sdha* (succinate dehydrogenase (ubiquinone) flavoprotein subunit) and *cyt b* (cytochrome b) were significantly downregulated at 0.5–1 μg/L of dinotefuran (DIN_2–DIN_3) (Figures 4B,C).

## 4 Discussion

Neonicotinoid insecticides are widely used as alternatives to traditional pesticides due to less toxic to mammals. They were usually detected in the environment (source water, tap water, fruit, and vegetable), even in human samples (16–19). Dinotefuran, as the third generation of neonicotinoid insecticides, was more safe for the environment and humans (3). However, our present study demonstrated that chronic (10 days) exposure to environmental concentrations (0.1–1 μg/L) of dinotefuran resulted in behavioral inhibition of the larvae, even death. Xiong et al. (6) reported that dinotefuran was detected with a mean concentration of 200 ± 296 ng/L and a maximum concentration of 802 ± 139 ng/L from a paddy field to receiving waters in the Poyang Lake basin of China. Meanwhile, dinotefuran was detected with a concentration of 12.7–75.5 ng/L in rivers near maize fields in Ontario, Canada (7) and 1.60–134 ng/L in streams across the United States (8). Our present report revealed that the LC_10_ values (0.38 (0.12–1.06) and 0.08 (0.02–0.33) μg/L) of dinotefuran after 8 and 10 days exposure to 1st instar larva of *C. kiiensis* were lower than the environmental concentrations (0.1–0.8 μg/L) (6, 8). Moreover, after 10 d exposure, the behavioral inhibition effect IC_50_ (0.60 (0.44–0.82) μg/L) was also lower than the environmental concentrations. These results suggested that choric exposure to dinotefuran in actual environment could cause lethality and paralysis to Chironomidae, even other aquatic organisms.

The toxicity target of neonicotinoids is the nAChRs of insects. First, they activate the nAChRs, then interfere with the central nervous system of insects, leading to overstimulation. Thus, insects become paralyzed and even die (11). Although the main modes of action (MoAs) of neonicotinoids to target species have been well characterized, numerous recent reports have found the unintended toxic effects of neonicotinoids on non-target organisms, even humans. Therefore, the exploration of their additional toxic mechanism has become the emerging focus of public attention. The nAChRs are pentameric ligand-gated ion channels selective for cations, including permeable to Ca^2+^. The entry of Ca^2+^ through nAChR channels has been demonstrated to regulate Ca^2+^-dependent cellular processes, such as the release of many neurotransmitters (20). Calcium is essential to adjust a large number of neuronal processes. Our present study demonstrated that dinotefuran enhanced Ca^2+^ influx via dysregulating the gene expressions of *atp2b*, *camk ii*, and *calm* at environmental concentrations. The gene *atp2b* is related to Ca^2+^ transporting ATPase plasma membrane. ATPase pumps played an important role in Ca^2+^ transporting (21). The gene *calm* is related to the protein Calmodulin is the predominant intracellular receptor of Ca^2+^ and is a highly conserved Ca^2+^ sensor, which is ubiquitously expressed in mammalian cells (22). The two genes *calm* and *camk ii* were involved in the synthesis of calcium ion regulatory proteins. Our previous studies also revealed that imidacloprid significantly interfered with the expressions of these genes and related proteins (15). Similarly, neonicotinoid insecticides (dinotefuran, nitenpyram, and acetamiprid) amplified Ca^2+^ influx via activating the store-operated Ca^2+^ entry (SOCE) in mice liver (23). Intracellular calcium signaling through nAChRs was activated by imidacloprid (10–100 μmol/L) in the dopaminergic Lund human mesencephalic (LUHMES) cell line (24). Imidacloprid overloaded Ca^2+^ influx by activating the nAChRs (25). However, most of the current studies did not take into account the reality of environmental concentrations, therefore most of the studies were conducted at high concentrations of acute exposure. Given this, we selected from low to high concentrations (0.1–1 μg/L) of dinotefuran for long-term exposure to chironomids. Our results revealed that dinotefuran under environmental concentrations (0.5–1 μg/L) and 10 d exposure, disrupted the calcium signaling pathway of midge by increasing the Ca^2+^ levels. Guzman-Vallejos et al. (26) revealed that 250–500 μmol/L of imidacloprid evoked more calcium changes in differentiated human neuroblastoma cells SH-SY5Y neurons by molecular docking analysis. Taha et al. (27) reported that neonicotinoid insecticides may be involved in a CaMKK/AMPK pathway in the regulation of neuron nAChRs, meanwhile, the calcium-calmodulin-dependent protein kinase inhibitor, STO-609, inhibited currents induced by neonicotinoids and the increase of intracellular calcium. To sum up, the genes, *atp2b*, *calm*, and *camk ii* of the calcium pathway might be the key biomarkers for dinotefuran.

The increase of intracellular Ca^2+^ was closely related to the overproduction of ROS. Our results suggested that dinotefuran increased ROS levels, along with the increase of H_2_O_2_ and MDA concentrations. ROS are highly active radicals formed upon unpaired electrons of oxygen (e.g., hydroxyl radical (•OH) and superoxide (•O_2_^−^)). Excessive ROS in biological systems could induce oxidative stress, which is closely related to physiological and pathological processes, such as aging and the development of cancer (28). When ROS levels exceed antioxidant defense capabilities, lipids, and proteins damage would be triggered, leading to lipid peroxidation (29). Accordingly, our study found that the MDA level of the midge, an indicator for lipid peroxidation, was significantly increased after exposure to dinotefuran. Meanwhile, the expressions of three key genes (*akt*, *cat*, and *sod*) of midge were altered exposed to above 0.1 μg/L of dinotefuran. A previous study reported the disorder of calcium signaling overproduced ROS concentration (30). Li et al. (23) reported that Ca^2+^ overload caused by dinotefuran was associated with the overproduction of ROS through the manipulation of SOCE protein expression because ROS scavenger n-acetylcysteine could attenuate Ca^2+^ overload induced by neonicotinoid insecticides. Previous reports found that AKT, as the key protein of oxidative stress, was also affected by neonicotinoid insecticides. For example, the phosphorylation of AKT (p-AKT) was reduced by imidacloprid in mice and human cells (31). Imidacloprid significantly decreased the ratio of p-AKT/AKT in the SH-SY5Y cells (15). Similarly, Wang et al. (38) revealed that intracellular ROS levels were markedly raised, and the activity of the cellular antioxidant enzymes (CAT, SOD, and GPx) was diminished when chicken lymphocyte lines were exposed to 110 μg/mL imidacloprid for 24 h.

The mitochondrion is a major producer of ROS in cells. In the present study, long-term exposure to dinotefuran reduced the levels of MMP and ATP of the larva, suggesting the mitochondrial dysfunction of the larva. Ca^2+^ overload is sufficient to induce the mitochondrial permeability transition (MPT), which is important in necrosis and apoptosis (32). The opening of mitochondrial membrane permeability transport pores can cause the breakdown of MMP. The reduction of MMP which was the key factor of mitochondrial homeostasis and oxidative phosphorylation, could induce the deficiency of ATP production. Mitochondrial damage can cause an imbalance between ROS production and removal, resulting in net ROS production. Inversely, increasing ROS or decreasing ATP necessary for repair, may exacerbate mitochondrial dysfunction (33). Some previous reports revealed that mitochondria are important neonicotinoid targets (34). After exposure to environmental-related concentrations (5 and 50 μg/L) of dinotefuran for 21 days, mitochondria fusion of *Xenopus laevis* tadpoles was excessively manifested and the mitochondrial respiratory chain was also disturbed, which brought about the rise of ROS production and a reduction of the ATP levels (35). Thus, cardiotoxicity associated with mitochondrial disorders was induced by dinotefuran. Neonicotinoid insecticides imidacloprid also led to mitochondrial damage via inhibiting FoF1-ATPase activity in rats (36). The concentrations of imidacloprid in the human urinary were significantly correlative with the mitochondrial DNA copy number, suggesting the possibility of dose-dependent mitochondrial damage (37). In the present study, the expressions of mitochondria-related genes of *atpef0*a, *sdha*, and *cyt b* were significantly downregulated in exposure groups, indicating the potential biomarkers for dinotefuran. These results suggested that dinotefuran disrupted the mitochondrial electron transport chains (ETCs). *In vitro* assays based on SH-SY5Y cells, neonicotinoid inhibited the expressions of these genes encoding mitochondrial oxidative phosphorylation complexes I and III (e.g., CytB, and CYC1), while increasing the production of ROS (37). Our previous study has revealed that imidacloprid significantly decreased the expression of important genes, *atpef0a/d*, *atpev1g*, and *cox2* related to the mitochondrial pathway in larvae of *C. dilutus* (15). Additionally, 110 mg/L imidacloprid upregulated the gene expressions of mitochondrial apoptosis (*Caspase 3*, *Caspase 9*, *Bax*, and *Cyt-c*) and necroptosis (*Caspase 8*, *RIPK1*, *RIPK3*, and *MLKL*) related factors of chicken lymphocyte lines (38). Thus, the disruption of the Ca^2+^ signal pathway and mitochondrial dysfunction disrupted the nerve system by interdicting normal neurotransmission, inhibiting behaviors, and eventually leading to the death of the midge.

**Figure 5:**
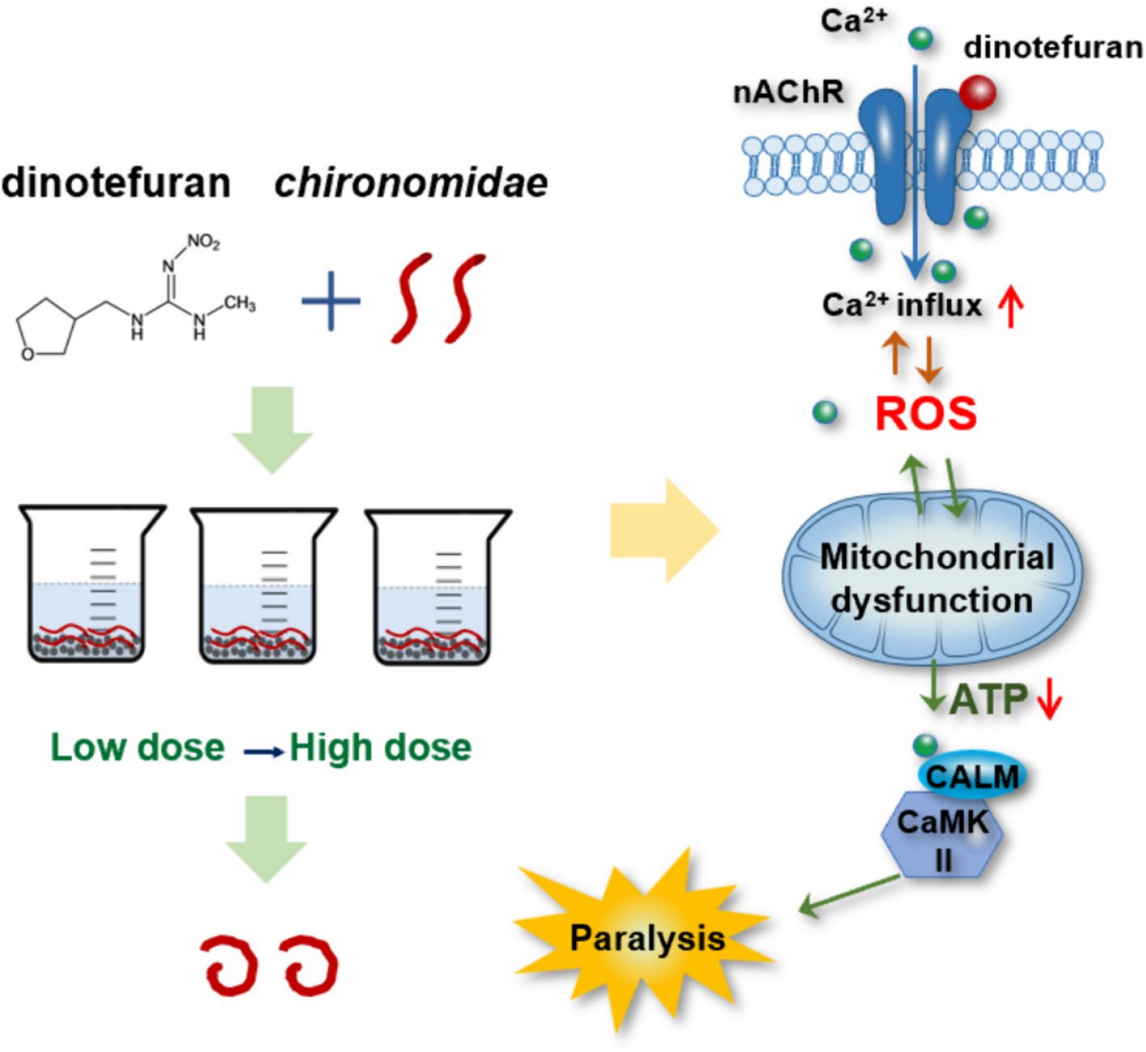
Toxicity pathway of dinotefuran acted on chironomid larvae.

In summary, calcium signaling was the key biomarker for dinotefuran. The excess Ca^2+^ influx led to the ROS overproduction. The interference of the Ca^2+^–ROS pathway would damage the mitochondrial function. Meanwhile, mitochondria are the sites of ROS production, the mitochondrial dysfunction would affect the production of ROS. Thus, the neurotransmission depended on Ca^2+^/calmodulin-mediated signal transduction was intercepted, triggering aberrant behaviors of the midge larva (Figure 5). Meanwhile, dysregulated genes (*atp2b*, *calm*, *camk ii*, *atpef0*a, *sdha*, and *cyt b*) in these response pathways at environmental concentrations may be important early biomarkers of dinotefuran.

## 5 Conclusion

The mechanism by which neonicotinoids cause behavioral inhibition and thus death in insects via acting on targets has been well known. However, the discovery of a large number of new toxicities, especially for non-target organisms, has stimulated the exploration of new toxicity mechanisms. In addition, most of the past toxicity reports were based on acute toxicity studies at high concentrations. However, the environmental concentrations of neonicotinoids are relatively low. At environmental concentrations, some studies have shown neonicotinoids negatively impact the health of aquatic organisms, even humans. Therefore, it is urgent to explore the mechanism of chronic toxicity at environmental concentrations. Our present study showed that long-term (10 days) exposure to environmental concentrations of dinotefuran resulted in behavioral inhibition of the larvae, even death of the Chironomidae larvae. Dinotefuran promoted the release of intracellular Ca^2+^ in Chironomidae. Subsequently, the disruption of the calcium signaling pathway induced oxidative stress by ROS overproduction, Thus, the over-release of Ca^2+^ and ROS disordered the mitochondrial-related pathway by dysregulating the expressions of mitochondria-related genes. Our findings showed low environmental concentrations of dinotefuran caused paralysis of the midge via interfering the Ca^2+^–ROS–mitochondria pathway. Dysregulated genes (*atp2b*, *calm*, *camk ii*, *atpef0*a, *sdha*, and *cyt b*) in these response pathways at environmental concentrations may be important early biomarkers of dinotefuran. However, more validation is necessary to support the current results. Calcium signaling and mitochondrial dysfunction were identified as the potential early warning responses for neonicotinoids, providing key biomarkers for aquatic risk assessment.

## Data Availability

All datasets generated for this study are included in the article/Supplementary material. More detailed data are available on request to the corresponding author.
