# Supplementary material for: Paralysis caused by dinotefuran at environmental concentration via interfering the Ca2+–ROS–mitochondria pathway in Chironomus kiiensis
Source: Front Public Health. 2024 Oct 2;12:1468384. doi: 10.3389/fpubh.2024.1468384 (PMC11479960; doi:10.3389/fpubh.2024.1468384)
Supplement: Supplementary file 1 [file Data_Sheet_1.docx]

**Supplemental Information**

Paralysis caused by dinotefuran at environmental concentration via interfering Ca^2+^-ROS-mitochondria pathway in *Chironomus kiiensis*

Fenghua Wei^a^, Weiwen Gu^a^, Fengru Zhang^a,*^, Shuangxin Wu^b,*^

^a^ School of Chemistry and Environment, Jiaying University, Meizhou 514015, China

^b^ School of Physics and Electrical Engineering, Jiaying University, Meizhou 514015, China

*Corresponding author

Fengru Zhang

School of Chemistry and Environment, Jiaying University, Meizhou 514015, China

E-mail: zhangfengru@126.com

Shuangxin Wu

School of Physics and Electrical Engineering, Jiaying University, Meizhou 514015, China

E-mail: [409794313@qq.com](mailto:409794313@qq.com)

**The file includes:**

Supporting Tables: 6

Supporting Figures: 1

References: Page S9

*Quantification of dinotefuran*

Exposure samples were collected from test containers at 5 and 10 days of the exposure. The sample was extracted using solid phase extraction (SPE) cartridges packed with 200 mg of HLB absorbent. Before loading the sample, the SPE cartridge was conditioned with 3 mL of methanol and 10 mL of water, sequentially. Samples were then passed through the cartridges at a flow rate of 3–5 mL/min and eluted out of the cartridges with 10 mL of methanol. Eluants were evaporated to near dryness under a gentle flow of nitrogen and re-dissolved in 50 μL of acetonitrile. The HPLC-MS/MS analysis was conducted on a Shimadzu DGU-30A HPLC coupled with an AB SCIEX TRIPLE QUAD™ 5500 tandem MS system. The analytes were separated on an Agilent Zorbax Eclipse Plus C18 column (100 × 2.1 mm i.d., 1.8 μm) at 40 ^o^C. The mobile phase was a mixture of water containing 0.1% formic acid (A) and acetonitrile (B) and the flow rate was 0.3 mL/min. The gradient elution condition was as follows: 0 min, 37% B; 1.2 min, 37% B; 3 min, 70% B; 3.5 min, 70% B; 3.6 min, 37% B; 5.1 min, 37% B. The injection volume was 2 μL. The MS monitoring was performed using an electrospray ionization (ESI) source in positive mode and multiple-reaction monitoring (MRM). The MS/MS conditions were as follows: source temperature, 550 ^o^C; curtain gas (CUR), 40 psi; collision gas (CAD), 7 psi; ion source gas1 (GS1): 55 psi; ion source gas 2 (GS2): 55 psi; ionspray voltage (IS), 5500 V; entrance potential (EP), 10 V; collision cell exit potential (CXP), 16 V. Other MS parameters for dinotefuran qualification are listed in **Table S1**. Quantification of dinotefuran was achieved using an internal standard calibration method and the calibration curve for dinotefuran was linear over a range of 0.1–100 μg/ L.

**Table S1.** Qualification parameters for analyzing dinotefuran, thiamethoxam-*d_3_* (internal standard), and imidacloprid-*d_4_* (surrogate standard) using HPLC-MS/MS.

| Compound | Transition mass (m/z) | Declustering Potential (V) | Collision energy (eV) | Retention time (min) |
| --- | --- | --- | --- | --- |
| Dinotefuran | 203.1→129.1^a^ | 50 | 16 | 2.23 |
|  | 203.1→114.0^b^ | 50 | 17 |  |
| Imidacloprid-*d_4_* | 260.1→179.1^a^ | 100 | 30 | 1.51 |
|  | 260.1→213.1^b^ | 100 | 22 |  |
| Thiamethoxam-*d_3_* | 295.1→214.0 ^a^ | 120 | 18 | 1.20 |
|  | 295.1→131.9 ^b^ | 120 | 30 |  |

^a^ MS/MS transition used for quantification

^b^ MS/MS transition used for confirmation

**Table S2.** Primers used in the real-time quantitative polymerase chain reaction (RT-qPCR).

| Function | Gene symbol | GenBank accession No. | Gene description | The size of product (bp) | Primer sequence (5′–3′) | Reference |
| --- | --- | --- | --- | --- | --- | --- |
| Internal reference | *β-actin* | AB070370 | *Chironomus yoshimatsui* mRNA for actin | 212 | F: GATGAAGATCCTCACCGAACG R: GGTTCATTACCGATTTGATG | (Wiseman et al., 2013) |
| Calcium influx | *atp2b* | — | Ca^2+^ transporting ATPase, plasma membrane | 198 | F: TAAACGCGGCGCACATTTAG  R: TTCTCGTCCCCTATGCTCCA | (Wei et al., 2020) |
|  | *camk ii* | — | calcium/calmodulin-dependent protein kinase (CaM kinase) II | 153 | F: TGTCATCAAAATGGGGTAGTTCA  R: CGTTCCAGCGAATCCAAACC | (Wei et al., 2020) |
|  | *calm* | — | calmodulin | 298 | F: AAAGGCGCCATCTCATTGGA  R: CCTCTGGCAATTCCGGATCA | (Wei et al., 2020) |
| Oxidative stress | *cat* | JL641904.1 | TSA: *Chironomus riparius* catalase mRNA | 100 | F: CGTGATCTTCGTGGTTTTGCTG R: GGATTGGATCGCGGATGAAG | (Nair et al., 2011) |
|  | *Cu/Zn-sod* | JQ342170.1 | *Chironomus riparius* copper-zinc superoxide dismutase (*Cu/Zn-sod*) mRNA | 81 | F: GTCGTGCTGTTGTCGTTCAT R: CAGCATTGCCAGTTTTGTGT | (Park et al., 2012) |
|  | *akt* | — | RAC serine/threonine-protein kinase | 119 | F: TGGACGTGGACGATCAACAG  R: GCTCAGATGGCTCCCTTGTT | (Wei et al., 2020) |
| Mitochondrial function | *atpef0a* | — | F-type H+-transporting ATPase subunit a | 216 | F: GATATTGCTAGGGTGGCGCT  R: GGGCGCAGTGATTATAGGCT | (Wei et al., 2020) |
|  | *sdha* | — | succinate dehydrogenase (ubiquinone) flavoprotein subunit | 175 | F: TTAAGGCTCGTCTCGCTTGG  R: TGTGTAGTCGTCGTTTCGCA | (Wei et al., 2020) |
|  | *cyt b* | AF109709.1 | Chironomus dilutus clone tent_LMA cytochrome b (cyt b) gene | 287 | F: TATGGGGCGGTTTTGCTGTA R: TGCTGGGATAAAATTATCAGGGTCT | (Wei et al., 2021) |

**Table S3.** The measured concentration of dinotefuran in exposure solution at 5 and 10 d (Data are expressed as mean ± standard deviation (n = 3)).

| Group^a^ | Nominal concentration (μg/L) | Measured concentration (μg/L) | |
| --- | --- | --- | --- |
|  |  | 5 d | 10 d |
| Negative control | 0 | ND | ND^a^ |
| Solvent control (0.1% DMSO) | 0 | ND | ND |
| DIN_1 | 0.1 | 0.08 ± 0.01 | 0.07 ±0.01 |
| DIN _2 | 0.5 | 0.70±0.04 | 0.64±0.03 |
| DIN_3 | 1 | 1.22 ±0.15 | 1.10 ±0.09 |
| DIN_4 | 5 | 4.61±0.16 | 4.73±0.11 |
| DIN_5 | 10 | 10.7±1.3 | 10.1±0.9 |
| DIN_6 | 50 | 47.2±1.4 | 46.8±6.2 |

^a^ ND: Not detected.

**Table S4.** Lethality (%) and burrowing inhibition (%) of the larva of *Chironomus kiiensis* after exposure to dinotefuran.

| Exposure group | Nominal concentration (μg/L) | Lethality (%, n=5) | | | | | | | | | | | | | | | | |
| --- | --- | --- | --- | --- | --- | --- | --- | --- | --- | --- | --- | --- | --- | --- | --- | --- | --- | --- |
|  |  | 4 d | | | | |  | 8 d | | | | |  | 10 d | | | | |
| Solvent control | 0 | 0 | 0 | 10 | 0 | 10 |  | 0 | 0 | 10 | 0 | 10 |  | 0 | 10 | 10 | 0 | 10 |
| DIN_1 | 0.1 | 0 | 10 | 0 | 10 | 0 |  | 10 | 10 | 0 | 10 | 0 |  | 20 | 20 | 10 | 20 | 10 |
| DIN _2 | 0.5 | 0 | 10 | 10 | 0 | 10 |  | 10 | 20 | 30 | 10 | 10 |  | 20 | 30 | 40 | 10 | 20 |
| DIN_3 | 1 | 10 | 20 | 30 | 20 | 0 |  | 20 | 20 | 50 | 20 | 20 |  | 30 | 30 | 60 | 30 | 20 |
| DIN_4 | 5 | 20 | 20 | 40 | 30 | 10 |  | 20 | 30 | 60 | 30 | 20 |  | 30 | 40 | 70 | 30 | 20 |
| DIN_5 | 10 | 50 | 60 | 20 | 40 | 20 |  | 60 | 60 | 30 | 40 | 30 |  | 70 | 60 | 30 | 40 | 50 |
| DIN_6 | 50 | 50 | 60 | 70 | 40 | 60 |  | 60 | 60 | 70 | 50 | 70 |  | 70 | 70 | 80 | 50 | 70 |
| Exposure group | Nominal concentration (μg/L) | Burrowing inhibition (%, n=5) | | | | | | | | | | | | | | | | |
|  |  | 4 d | | | | |  | 8 d | | | | |  | 10 d | | | | |
| Solvent control | 0 | 0 | 0 | 10 | 0 | 10 |  | 0 | 0 | 10 | 0 | 10 |  | 0 | 10 | 10 | 0 | 10 |
| DIN_1 | 0.1 | 10 | 10 | 20 | 10 | 0 |  | 10 | 30 | 30 | 30 | 20 |  | 20 | 40 | 40 | 30 | 40 |
| DIN _2 | 0.5 | 0 | 10 | 20 | 0 | 10 |  | 30 | 20 | 30 | 50 | 20 |  | 30 | 40 | 40 | 50 | 30 |
| DIN_3 | 1 | 10 | 20 | 30 | 20 | 0 |  | 20 | 60 | 50 | 30 | 20 |  | 70 | 60 | 70 | 50 | 80 |
| DIN_4 | 5 | 20 | 20 | 40 | 30 | 50 |  | 90 | 80 | 60 | 90 | 60 |  | 90 | 90 | 70 | 80 | 90 |
| DIN_5 | 10 | 80 | 80 | 50 | 40 | 50 |  | 80 | 100 | 90 | 90 | 80 |  | 90 | 90 | 100 | 100 | 100 |
| DIN_6 | 50 | 60 | 60 | 90 | 50 | 70 |  | 90 | 100 | 100 | 80 | 100 |  | 100 | 100 | 100 | 100 | 100 |

**Table S5.** Ca^2+^, ROS, H_2_O_2_, MDA, MMP, and ATP levels in 1st instar larva after exposure to dinotefuran until the first pupa appears. Data are expressed as mean  ±  standard error (n = 3). (H_2_O_2_, hydrogen peroxide; MDA, malondialdehyde; MMP, mitochondrial membrane potential; ATP, adenosine triphosphate)

| Group | Ca^2+^ | ROS | H_2_O_2_ | MDA | MMP | ATP |
| --- | --- | --- | --- | --- | --- | --- |
| Control | 1.00±0.05 | 1.00±0.05 | 1.00±0.08 | 1.00±0.11 | 1.00±0.09 | 1.00±0.06 |
| DIN_1 | 1.03±0.09 | 1.19±0.05 | 1.55±0.09 | 1.59±0.08 | 1.06±0.03 | 0.95±0.06 |
| DIN_2 | 1.55±0.14 | 1.49±0.12 | 1.24±0.19 | 1.63±0.11 | 0.82±0.03 | 0.78±0.08 |
| DIN_3 | 1.65±0.22 | 1.28±0.09 | 1.64±0.24 | 1.87±0.22 | 0.62±0.05 | 0.74±0.03 |

**Table S6.** Genes expressions in 1st instar larva after exposure to dinotefuran until the first pupa appears. Data are expressed as mean ± standard error (n = 3).

| Group | *atp2b* | *camk ii* | *calm* | *cat* | *sod* | *akt* | *atpef0a* | *sdha* | *cy b* |
| --- | --- | --- | --- | --- | --- | --- | --- | --- | --- |
| Control | -0.06±0.11 | 0.00±0.11 | 0.00±0.06 | -0.02±0.18 | 0.00±0.06 | 0.00±0.06 | 0.02±0.09 | -0.02±0.04 | 0.00±0.04 |
| DIN_1 | 0.23±0.05 | 0.71±0.08 | -0.12±0.03 | 0.62±0.12 | 0.09±0.15 | -0.39±0.06 | -0.49±0.10 | -0.23±0.04 | 0.08±0.18 |
| DIN_2 | 0.87±0.17 | 0.72±0.10 | 1.10±0.19 | 1.48±0.20 | 1.05±0.14 | -0.67±0.11 | -0.64±0.08 | -0.60±0.11 | -0.63±0.04 |
| DIN_3 | 1.55±0.17 | 1.39±0.31 | 0.77±0.06 | 1.44±0.17 | 1.22±0.19 | -0.68±0.14 | -1.06±0.15 | -0.80±0.18 | -0.60±0.08 |


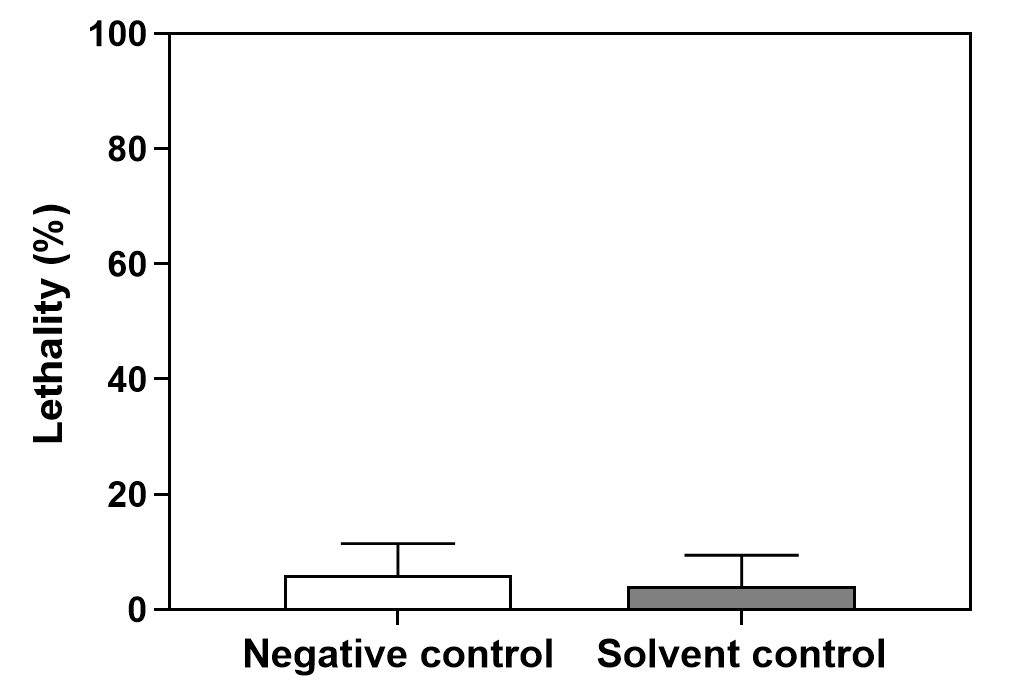


**Figure S1.** Lethality of negative control and solvent control of 1^st^ instar larva of *Chironomus kiiensis* on 10 d. *Data are expressed as mean ± standard error (n = 5).* *The asterisk denotes a significant difference between the two controls (p < 0.05).*
